# Abnormal gait and motor cortical processing in drug‐resistant juvenile myoclonic epilepsy

**DOI:** 10.1002/brb3.2872

**Published:** 2023-01-05

**Authors:** Inbal Maidan, Mor Yam, Sigal Glatt, Shai Nosatzki, Lilach Goldstein, Nir Giladi, Jeffrey M Hausdorff, Anat Mirelman, Firas Fahoum

**Affiliations:** ^1^ Brain Electrophysiology and Epilepsy Lab, Epilepsy Unit, Neurological Institute Tel Aviv Sourasky Medical Center Tel Aviv Israel; ^2^ Laboratory of Early Markers of Neurodegeneration, Center for the Study of Movement, Cognition and Mobility, Neurological Institute Tel Aviv Sourasky Medical Center Tel Aviv Israel; ^3^ Sackler Faculty of Medicine Tel Aviv University Tel Aviv Israel; ^4^ Sagol School of Neuroscience Tel Aviv University Tel Aviv Israel; ^5^ Sackler Faculty of Medicine Department of Physical Therapy Tel Aviv University Tel Aviv Israel; ^6^ Rush Alzheimer's Disease Center and Department of Orthopedic Surgery Rush University Medical Center Chicago Illinois

**Keywords:** current source densities (CSD), drug‐resistant (DR), dual‐task, gait, juvenile myoclonic epilepsy (JME)

## Abstract

**Background:**

Juvenile myoclonic epilepsy (JME) is characterized by generalized seizures. Nearly 30% of JME patients are drug‐resistant (DR‐JME), indicating a widespread cortical dysfunction. Walking is an important function that necessitates orchestrated coordination of frontocentral cortical regions. However, gait alterations in JME have been scarcely investigated. Our aim was to assess changes in gait and motor‐evoked responses in DR‐JME patients.

**Methods:**

Twenty‐nine subjects (11 JME drug‐responder, 8 DR‐JME, and 10 healthy controls) underwent a gait analyses during usual walking and dual‐task walking. Later, subjects underwent 64‐channel EEG recordings while performing a simple motor task. We calculated the motor‐evoked current source densities (CSD) at a priori chosen cortical regions. Gait and CSD measures were compared between groups and tasks using mixed model analysis.

**Results:**

DR‐JME patients demonstrated an altered gait pattern that included slower gait speed (*p* = .018), reduced cadence (*p* = .003), and smaller arm‐swing amplitude (*p* = .011). The DR‐JME group showed higher motor‐evoked CSD in the postcentral gyri compared to responders (*p* = .049) and both JME groups showed higher CSD in the superior frontal gyri compared to healthy controls (*p* < .011). Moreover, higher CSD in the superior frontal gyri correlated with worse performance in dual‐task walking (*r* > |–0.494|, *p* < .008).

**Conclusions:**

These alterations in gait and motor‐evoked responses in DRE‐JME patients reflect a more severe dysfunction of motor‐cognitive neural processing in frontocentral regions, leading to poorer gait performance. Further studies are needed to investigate the predictive value of altered gait and cortical motor processing as biomarkers for poor response to treatment in JME and other epilepsy syndromes.

## Graphical abstract

Patients with JME demonstrated abnormal gait pattern compared to healthy controls, which were even more accentuated in drug‐resistant patients. Furthermore, we found different spatial distribution of motor evoked potentials that strongly correlated with the abnormal gait measures. Overall, our results suggest that detailed gait assessment should be sought after in JME patients, as it possible reflects motor‐cognitive neural integrity and patients' responsiveness to medications.

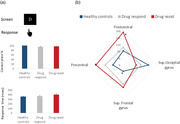

## 1 INTRODUCTION

Juvenile myoclonic epilepsy (JME) is the most common genetic generalized epilepsy syndrome, accounting for 10% of all epilepsies (Camfield et al., 2013). It is characterized by bilateral motor (myoclonic) jerks and other generalized seizure types, cognitive disturbances such as impaired attention and difficulties in inhibiting habitual responses (Smith et al., 2020; Wolf et al., 2015). Electroencephalography (EEG) recordings in JME patients show generalized epileptiform discharges that are maximal frontocentrally (Genton et al., 2013). The pathophysiology of JME and the associated motor and cognitive symptoms are postulated to originate from dysfunctional frontocentral‐thalamic circuitries (Gloor & Fariello, 1988; Patrikelis et al., 2020). EEG as well as structural and functional neuroimaging studies support this notion, pointing to abnormal frontocentrothalamic circuitries (Kazis et al., 2021; Roebling et al., 2009).

Most JME patients respond well to antiseizures medications (ASMs), yet up to 30% of patients continue to experience seizures despite treatment with appropriate ASMs and suffer from drug‐resistant JME (DR‐JME) (Geithner et al., 2012; Gelisse et al., 2001). Compared to drug‐responder patients, DR‐JME patients present more cognitive impairments. Neuroimaging studies show disrupted brain networks that correlate with the severity of cognitive deficits in DR‐JME patients (O'Muircheartaigh et al., 2011; Pulsipher et al., 2009). Altogether, these clinical, electrophysiological and neuroimaging findings portray more severe frontal networks dysfunction in DR‐JME. Yet, DR‐JME is identified retrospectively, after failing a number of ASMs with continuing seizures and potential ASM adverse effects that increase morbidity and mortality and reduce quality of life (Dalic & Cook, 2016; Loscher et al., 2020). To date, there are no good biomarkers for DR‐JME.

Gait is a basic human behavior, yet it is far from being an automatic motor process. It is a complex task that necessitates orchestrated coordination of various frontocentral brain regions coalescing to motor‐cognitive functional neural networks. There is a growing body of evidence that walking is tightly controlled by higher cognitive processes that incorporate sensory information with motor adaptation to enable ambulation during everyday life conditions (Montero‐Odasso et al., 2012). The cognitive processing needed for gait is even more accentuated when gait is tested together with a cognitively demanding task, also known as dual‐task walking (Yogev‐Seligmann et al., 2008). The performance of dual‐task walking is related to cognitive abilities and reserves, especially in the domains of executive ‘frontal’ functions such as attention and inhibition (Montero‐Odasso et al., 2012; Yogev‐Seligmann et al., 2008). In a recently published study by our group, we recorded event related potentials (ERPs) while responder JME and DR‐JME patients performed visual Go/NoGo task while walking on a treadmill. We found a reduced ability to adjust N2 potential in patients with DR‐JME, reflecting abnormal frontal inhibition mechanism that is normally active during abstaining of motor action (during the NoGo trials) (Yam et al., 2022). Furthermore, this attenuation in N2 potential during dual‐task walking in DR‐JME patients was maximal in the central head regions, pointing to abnormal cognitive processing in networks that involve these regions (Yam et al., 2022).

Therefore, the aims of this study were (1) to assess gait performance during overground simple (single‐task) and dual‐task walking in responder and in DR‐JME patients and (2) to explore electrophysiological changes that relate to gait performance in JME patients. As JME is hypothesized to entail frontal motor‐cognitive dysfunction, we hypothesized that dual‐task walking will be altered in JME patients compared to healthy subjects, and specifically would be more disrupted in DR‐JME patients compared to responder patients. From an electrophysiological perspective, we postulated that frontocentral motor evoked responses and cognitive processing would be spatiotemporally altered in JME patients, and even more so in DR‐JME patients.

## 2 METHODS

### 2.1 Participants

Twenty‐nine subjects, 19 with JME (11 responders and 8 DR‐JME) and 10 healthy controls participated in this study. JME patients were prospectively recruited from the Epilepsy unit at Tel‐Aviv Sourasky Medical Center if they were >18 years and satisfied the clinical criteria for JME. Drug resistance was determined according to the DR definition of the International League against Epilepsy (ILAE) as “failure of adequate trials of two tolerated, appropriately chosen and used ASMs schedules to achieve sustained seizure freedom” (Kwan et al., 2010). Patients were tested on their regular ASM regimen (Supplementary Table S1). Age and gender matched healthy controls without any neurological or psychiatric disorder were included. The study was approved by the local ethical committee according to the principles of the declaration of Helsinki. All participants gave their informed written consent prior to participation.

### 2.2 Procedures

Eligible participants underwent clinical and cognitive evaluations, detailed gait assessment, and a high‐density 64‐channel EEG recording while performing a simple motor task of pressing a button. The clinical data were collected from patients’ charts and subjects were further asked to fill in personal characteristic's questionnaire, disease characterization and medications for JME patients, and a quality of life questionnaire (Tefera et al., 2020). The cognitive evaluation included the Montreal Cognitive Assessment (MoCA) which provides a global cognitive function assessment (Nasreddine & Patel, 2016), and the Color Trail Test (CTT), which evaluates visual scanning, attention, inhibition, and cognitive flexibility (Sanchez‐Cubillo et al., 2009).

The gait assessment consisted of walking in a 10‐m corridor for 1 min under two walking conditions: (1) usual walking at the preferred walking speed and (2) dual‐task walking while simultaneously performing a cognitive task of serial 7 substractions with no explicit task prioritization. The walking tasks were performed while subjects had five precalibrated and synchronized body fixed sensors (OpalAPDM, Portland, OR, USA) with triaxial accelerometers and gyroscopes positioned on the participant's lower back (level of L4−5 spine), right and left ankles and wrists to quantify gait and arm swing (Mirelman et al., 2021).

The EEG recording was performed while the participants did a simple motor task seated comfortably in front of a computer screen. The simple motor task was extracted from well‐known classical visual Go‐NoGo task, in which the participants were required to press the keypad with their dominant hand as quickly and as accurately in the appearance of GO cue. The Go cue consisted of white English letters appearing over a black background in the center of a computer screen, between two vertical white lines which remain on the screen throughout the whole duration of the experimental. The task included two sessions, each of 6 min that comprised 400 cues (Yam et al., 2022). The interstimulus interval was randomly varied between 1000 and 3000 ms with steps of 250 ms. All the correct responses and their response time were used for further analysis.

### 2.3 Gait analyses

The movement signals recorded via the accelerometers were filtered using a low‐pass Butterworth filter with a frequency cut‐off of 3.5 Hz with a band‐pass of less than 0.5 db, as previously described (Hillel et al., 2019). Turns during the two walking conditions were identified from the yaw movement around the vertical axis obtained from the gyroscope and removed from the analysis. The gait measurements included spatiotemporal features as gait speed, cadence (the number of steps per minute), stride time CV (variability between strides time), and arm‐swing amplitude.

### 2.4 EEG recording and preprocessing

EEG was recorded using a 64‐channels system (GES 400, EGI, USA) positioned according to the international 10‐10 system. Electrooculogram (EOG) signals were recorded using four channels—two placed above and below the right eye and two at the outer canthi of both eyes. EEG and EOG were recorded with a sampling rate of 250 Hz and the reference electrodes were positioned bilaterally over the mastoid bones behind the ears. The EEG data were preprocessed by the EEGLAB open‐source MATLAB software package. First, the data was passband filtered using a zero‐phase hamming windowed sync FIR filter between 0.1−40 Hz in order to discard low and high bands artifacts. Channels with remaining prominent artifacts were removed based on visual inspection. Next, the data were re‐referenced to a common average montage (average of all scalp electrodes) and independent component analysis (ICA) was applied to the dataset to remove EOG and EMG artifacts.

### 2.5 Calculation of motor‐evoked current source density (CSD)

After EEG preprocessing, we extracted epochs of correct motor responses. The response time to the GO cue reflected the motor action of pressing the keypad. Therefore, we averaged all the responses time to the GO cue and calculated the mean response time (motor action) for each participant. Since the average motor response time of all the participants was 320 ms and the standard deviation was 112 ms, we selected a window of 100 ms, 50 ms before and after the average time, to include most of the motor actions. These 100 ms time windows were used to calculate the current source density (CSD) in specific brain regions that were chosen a priori. The use of CSD, the Laplacian of the scalp surface voltage, to map the sources of electrical activity of the brain is a powerful method that is being increasingly used in cognitive and affective studies (Kamarajan et al., 2015). Moreover, CSD and Laplacian measures have proven effective in elucidating topographic and activation differences between neuropsychiatric conditions such as schizophrenia, depression, anxiety disorders, and neurological conditions (i.e., epilepsy) (Kamarajan et al., 2015).

We used LORETA software (version 2020) (Herrmann et al., 2004; Pascual‐Marqui et al., 2002) in order to calculate CSD (Kayser & Tenke, 2015; Rangel‐Gomez et al., 2015). This method transforms scalp‐recorded EEG into estimates of radial current flow at scalp (Kayser & Tenke, 2015), and by that it provides effective topography that reduces the negative impact of volume conduction and enhances the spatial resolution of the EEG signal. In addition, CSD does not require previous assumptions regarding structural and functional neuroanatomy (Kayser & Tenke, 2015; Rangel‐Gomez et al., 2015). A priori we chose to examine the CSD in four regions of interest (ROI), bilaterally: the postcentral gyri representing the primary sensory cortices, the precentral gyri representing the primary motor cortices, the superior frontal gyri representing a cognitive processing region, and the superior occipital gyri as secondary visual brain areas that were used as control regions. These ROIs were chosen since they are pivotal to motor and cognitive functions, and even more so to gait control. Therefore, the examination of these brain areas allowed us to test our hypothesis, in which functions that rely on information processing in these frontocentral regions are impaired in JME patients. Given the generalized nature of JME and the bilateral and synchronous seizures in this syndrome, we assumed equal involvement of both hemispheres, and therefore we calculated the CSD as an average of both sides. Spatial data were coregistered and applied to a MNI305 digitized structural MRI template with a 5 mm resolution (Kayser & Tenke, 2015; Rangel‐Gomez et al., 2015).

### 2.6 Statistical analysis

The mean and standard deviation of the demographic variables were calculated and evaluated for normality and homogeneity using Kolmogorov–Smirnov tests. Kruskal–Walls and one‐way ANOVA, followed by Least Significant Difference (LSD) post hoc tests, were used to examine differences between groups in demographics and CSD measures. Linear‐mixed models were used to examine measures of gait while controlling for gender, age, and number of medications. The effect of group, condition (usual vs. dual‐task), and their interactions were tested. The correlations between motor‐cognitive measures and CSD measures were examined using Spearman's tests, setting significance level to *p* = .05. The statistical analyses were performed using SPSS statistics 27 software for Windows 2016.

## 3 RESULTS

### 3.1 Participants

The demographic and clinical characteristics of the patients and control groups are presented in Table 1. DR‐JME patients had less years of education and showed worse cognitive performance manifested as lower MOCA score, prolonged CTT‐B time, and smaller number of 7‐subtractions during walking task, as compared to healthy controls. Differences between drug‐responder and DR‐JME patients were found only in the number of currently used ASMs, with DR‐JME patients using more medications than drug‐responder JME patients. A complete list of the ASMs can be found in Supplementary Table S1. It is important to note that no significant difference in the VPA daily dose was found between the responders and DR‐JME patients (*p* = .793) (Table 1). More specifically, 5 of 11 responders (45%) were on 750–2000 mg VPA daily and 4 of 8 nonresponders (50%) were on 1250–1500 mg VPA daily (Supplementary Table S1).

**TABLE 1:** Participants’ characteristics (drug‐responders, drug‐resistant [DR‐JME], healthy control)

	Variable (mean ± SE)	Healthy (*n* = 10)	Drug‐responder (*n* = 11)	DR‐JME (*n* = 8)	*p* Values
**Demographic**	Age (years)	29.5 ± 0.7	31.5 ± 1.5	27.3 ± 2.3	.157
	Gender (M/F)	6/4	4/7	3/5	.658
	Years of education (years)	17.9 ± 0.9	15.0 ± 0.9	13.6 ± 0.7**	.008
**Cognitive**	MOCA	28.4 ± 0.6	26.3 ± 0.6	23.4 ± 1.4**	.001
	CTT (B‐A) (sec)	26.7 ± 5.1	38.6 ± 7.5	70.4 ± 17.8**	.023
	Serial 7 number of subtractions	15.4 ± 1.3	9.5 ± 1.9	5.2 ± 2.4**	.008
**Epilepsy characteristics**	Epilepsy duration (years)	–	9.6 ± 1.9	15.4 ± 3.1	.118
	Number of current ASMs	–	1.8 ± 0.3	3.0 ± 0.4^#^	.030
	Daily VPA dose (mg)	–	590.9 ± 687.5	808.3 ± 741.0	.793

DR‐JME = drug‐resistant juvenile myoclonic epilepsy, M = male, F = female, MOCA = montreal cognitive assessment, CTT = color trail test, ASM = antiseizures medications, VPA = valporate.

^*^Significant difference between healthy controls and drug‐responders.

** Significant difference between healthy controls and DR‐JME.

^#^ Significant difference between drug‐responders and DR‐JME.

### 3.2 Differences in gait measures between groups and conditions

**FIGURE 1:**
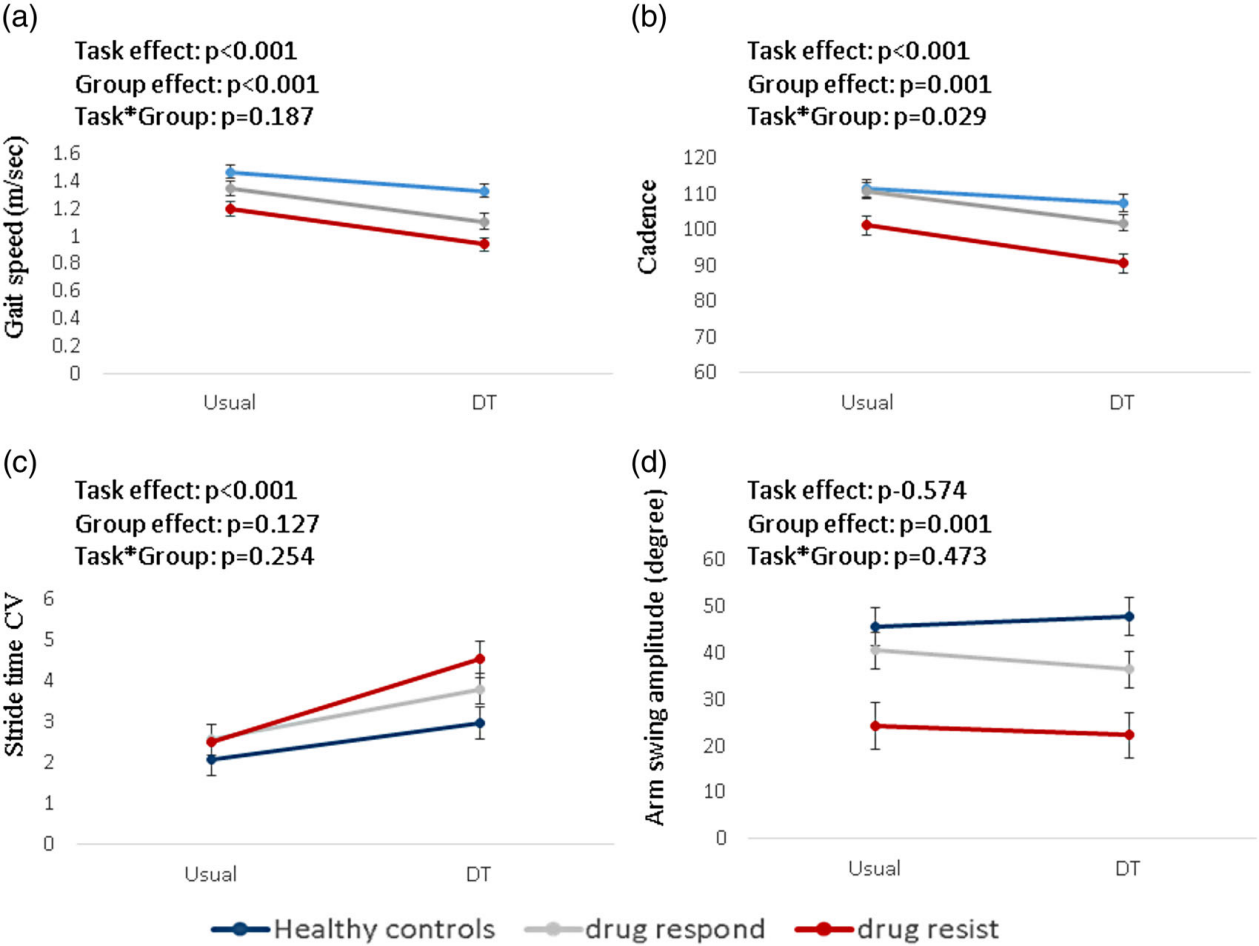
The effects of group and condition on gait measures. (a) Gait speed, (b) cadence, (c) stride time CV, and (d) arm swing amplitude. The error bars represent the standard errors

A significant effect of condition was found in gait speed, cadence, and stride‐time CV in all groups: Slower gait speed, lower cadence, and higher stride‐time CV (worse gait performance) were observed during dual‐task compared to usual walking (Figure 1). Significant effect of group was observed in gait speed, cadence, and arm swing amplitude (Figure 1). The number of medications did not show significant effect on these findings (gait speed: *p* = .338, cadence: *p* = .840, arm swing: 0.116). Specifically, DR‐JME patients demonstrated slower gait speed, reduced cadence, and smaller arm swing amplitude compared to drug‐responder JME patients (*p* = .024, *p* = .003, *p* = .011 respectively) and compared to healthy controls (*p* < .001). No significant differences between drug‐responder JME patients and healthy controls were found in stride‐time CV (*p* = .153), cadence (*p* = .325), and arm swing amplitude (*p* = .109). Gait speed was significantly slower in drug‐responders compared to healthy controls (*p* = .013). Significant interactions between condition and group were found in cadence (*p* = .029), showing larger differences between the groups during dual‐task walking (Figure 1b).

### 3.3 Group differences in motor‐evoked CSD

**FIGURE 2:**
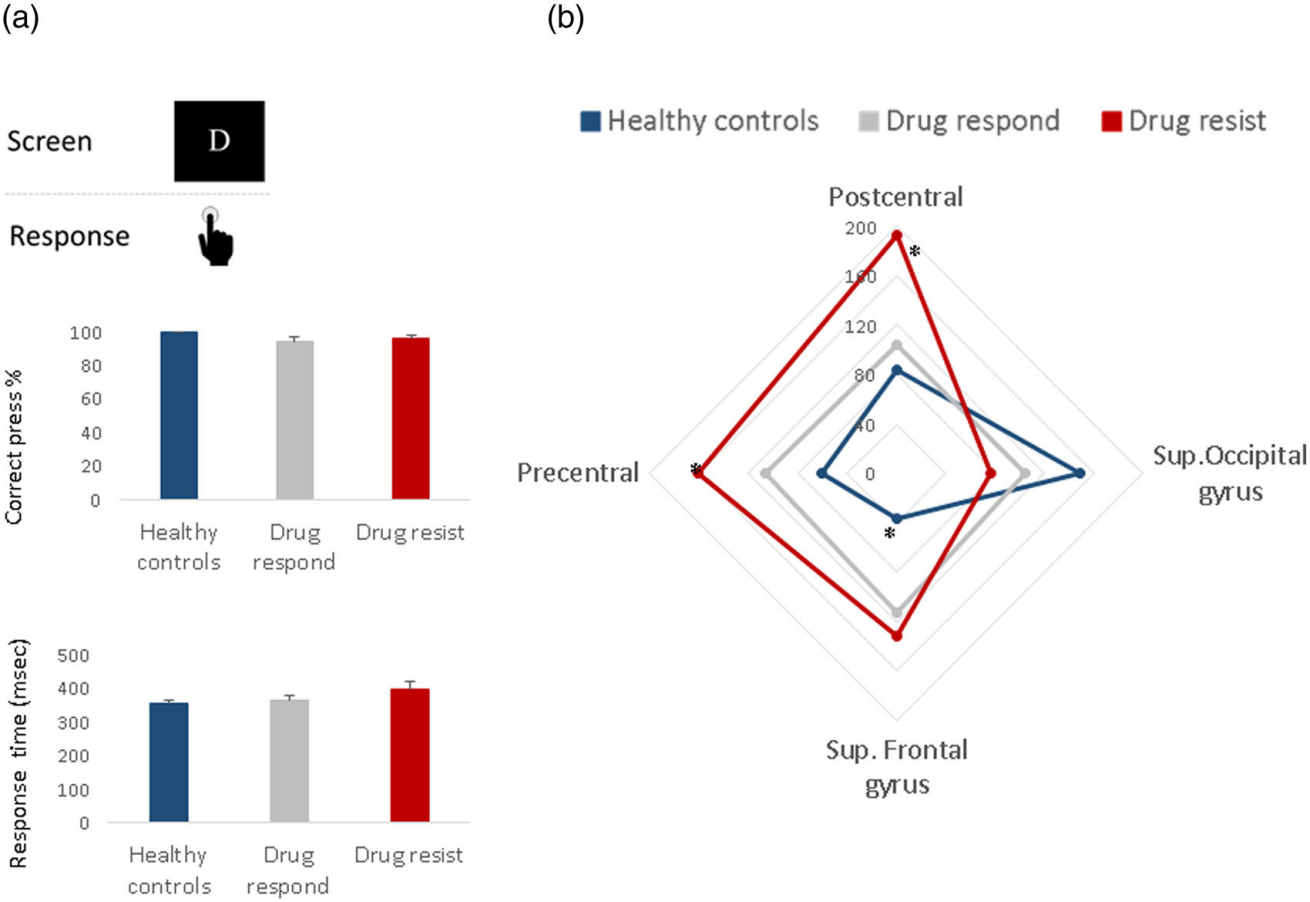
(a) The motor task performance measured as correct press % and response time and (b) CSD in four brain regions in each group. *p < .05

The percent of correct motor responses and response time did not differ between the groups (*p* = .128, *p* = .210, Figure 2a). In contrast, different CSD were found between the groups in the postcentral gyri (*p* = .050), precentral gyri (*p* = .046), and superior frontal gyri (*p* = .008) (Figure 2b). The number of medications did not show significant effect on these findings (postcentral gyri: *p* = .115, precentral gyri: *p* = .149, sup. frontal gyri: 0.662). DR‐JME patients showed higher CSD in the postcentral gyri compared to drug‐responder patients (*p* = .049) and healthy controls (*p* = .021). The CSD in the precentral gyri was significantly higher in DR‐JME patients compared to healthy controls (*p* = .014) and the CSD in the superior frontal gyri was significantly higher in both JME groups compared to healthy controls (drug‐responder vs. healthy controls: *p* = .011, DR‐JME vs. healthy controls: *p* = .004). In contrast, no significant differences between the groups were observed in the superior occipital gyri (*p* > .220).

### 3.4 Correlations between CSD and gait measures

**FIGURE 3:**
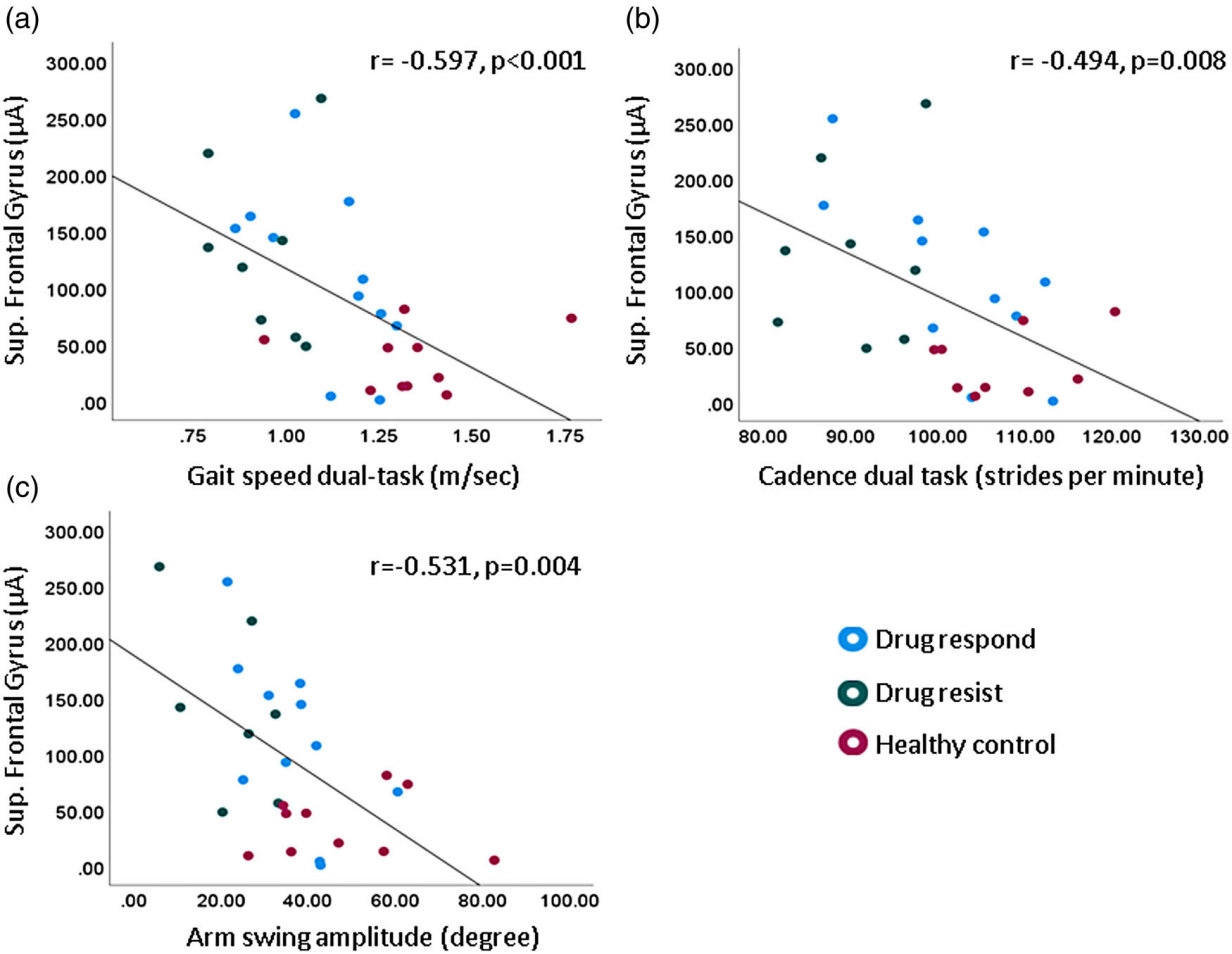
Correlations between CSD in superior frontal gyri and gait measures during dual‐task (a) gait speed, (b) cadence, (c) arm swing amplitude in all participants

We found significant correlations between motor‐evoked CSD in the superior frontal gyri and measures of gait during dual‐task (Figure 3). Higher motor‐evoked CSD in the superior frontal gyri correlated with worse performance in dual‐task walking measured by slower gait speed (*r* = −0.597, *p* < .001, Figure 3a) reduced cadence (*r* = −0.494, *p* = .008, Figure 3b), and smaller arm swing amplitude (*r* = −0.531, *p* = .004, Figure 3c). No significant correlations with usual walking measures were observed (*p* > .190). In addition, we did not find correlations between the other ROIs and usual and dual‐task walking measures (*p* > .117).

## 4 DISCUSSION

In this study, we assessed gait during simple and dual‐task walking, in drug‐responder and DR‐JME patients. In addition, we tested the CSD activity during a simple motor task in specific ROIs hypothesized to be affected in JME. Our results reveal four main findings: (1) DR‐JME patients have worse gait than drug‐responder JME patients and healthy controls in usual and dual‐task walking, (2) DR‐JME patients have higher motor‐evoked CSD in the postcentral gyri compared to responder JME patients and healthy controls, (3) both groups of JME patients demonstrated higher CSD activity in the superior frontal gyri compared to healthy controls (4) the motor‐evoked CSD in the superior frontal gyri correlated with dual‐task gait performance. It is important to note that the larger amount of medications in the DR‐JME patients did not have significant effects on these findings, indicating that the worse gait performance and the higher motor‐evoked CSD in DR‐JME patients were not just a result of larger amount of medications. In addition, the similar doses of VPA between the groups indicate that the gait differences cannot be attributed to the potential effects of VPA.

Gait assessment is a powerful tool that has been shown to predict the development of motor and cognitive decline (Hausdorff & Buchman, 2013) as well as survival in older adults (Studenski et al., 2011). Specific changes in gait have been described in many different neurological diseases (Hausdorff & Buchman, 2013; Montero‐Odasso et al., 2012; Moon et al., 2016), suggesting that gait impairments can be a reflection of neural dysfunction. Nevertheless, gait is not commonly and routinely studied in epilepsy patients, and has not been systematically assessed in JME patients. This study is the first to describe gait pattern alterations in JME. Furthermore, this aberrant gait pattern is even more pronounced in DR‐JME patients with observed slower gait speed, reduced number of steps per minute, and smaller arm‐swing amplitude compared to drug‐responder JME patients and healthy controls. In contrast, we did not observe changes in stride‐to‐stride variability between the groups, another common finding in extrapyramidal disorders (Giladi et al., 2013). Altogether, these observations suggest that JME patients have a unique gait pattern that could be used in clinical practice and in research, and this could provide valuable knowledge on disease characterization.

Studies have reported the impact of dual‐task on gait, even in healthy young adults. This likely stems from the increase in cognitive demands and reliance on executive functions and attention (Hausdorff et al., 2008; Srygley et al., 2009). In line with these studies, we found worse gait performance during dual‐task compared to usual walking in all groups. However, the group of DR‐JME patients showed the largest gait deterioration during dual‐tasking, manifesting with larger reduction in gait speed and cadence. The marked cognitive impairments reported in DR‐JME patients (Smith et al., 2020; Wolf et al., 2015) may explain the larger effects of dual‐task on gait. Gait is controlled by higher cognitive processes that involve complex neural networks (Hausdorff et al., 2008; Montero‐Odasso et al., 2012). Therefore, the worse gait pattern observed in DR‐JME patients already during simple walking and further under challenging condition reflects altered neural processing that encompasses motor and cognitive networks in JME. In addition, it may reflect a more severe neural dysfunction in DR‐JME, in comparison to responder patients.

To better understand the underlying neural mechanisms of these gait alterations in JME patients, and more specifically in DR‐JME patients, we measured the CSD during button press response to visual stimuli in specific ROIs. These ROIs are key nodes for sensory‐motor integration and frontal cognitive processing known to be affected in JME. The use of CSD, the Laplacian of the scalp surface voltage, to map the electrical activity of the brain during specific motor or cognitive task is a powerful method to identify the neuronal generator patterns contributing to scalp‐recorded EEG (Tenke & Kayser, 2012). These CSD maps represent the magnitude of the transcranial current flow from the brain to the scalp and from the scalp to the brain, corresponding to the positive and negative ERP activity (Herrmann et al., 2004; Tenke & Kayser, 2012). In fact, the CSD transformation functions as a high‐pass spatial filter that minimizes the electrical distortions produced by the mediums between cortical surface and EEG electrode such as skull and scalp, thus facilitating spatial separation of temporally overlapping components (Turetsky et al., 2000). Therefore, a brain region showing higher CSD during a specific task indicates that it is a stronger source for the elicited ERP, while a brain region showing lower CSD reflects a weaker source (Kamarajan et al., 2015).

We found CSD changes in regions that encompass sensory‐motor and cognitive processing in JME patients. Specifically, DR‐JME patients had higher CSD in the sensory‐motor regions that may relate to the gait alterations observed in this group. Moreover, a significant difference in which DR‐JME patients had higher CSD compared to responders was found only in the postcentral gyri, indicating alterations in sensory nodes. These findings portray different interconnected nodes with different patterns of motor activation that differ between the groups. In addition, we found higher CSD in the superior frontal gyri in both JME groups compared to controls, supporting the involvement of frontal networks in JME and the cognitive deficits associated with JME. No differences in CSD in the superior occipital gyri, a brain area that represents secondary visual processing, were found between the groups, indicating that the activation of these networks in response to task is preserved in JME and DR‐JME. Altogether, these changes in CSD emphasize the involvement of frontocentral networks in JME that are more pronounced in DR‐JME.

It is of note that the motor task performance, in response to a visual stimuli, did not differ between the groups, with all groups showing similar response time to stimuli. In contrast, the worse gait performance shown in DR‐JME patients reveal that complex tasks that require multiple resources may exceed capacity and affect performance. Interestingly, we found that worse dual‐task walking correlated with higher CDS in the superior frontal gyri. These findings are in line with the vast literature showing the important role of cognitive processing in walking while dual‐tasking. The lower cognitive performance in MOCA and CTT in DR‐JME patients and their worse gait emphasize the crucial interplay between motor and cognitive networks during walking in these patients.

This exploratory study has two main limitations. (1) The small sample size limits the generalization of the results and future studies with larger sample sizes are needed. Yet, the significant differences in various measures of gait and CSD in sensory, motor and cognitive brain regions between drug‐responder and DR‐JME patients provide interesting novel findings in this relative small cohort, which argue for the validity of our results. (2) JME patients were all treated with ASMs at the time of the study, and furthermore, DR‐JME patients used higher number of ASMs as compared to drug‐responder patients. The variability in the ASM regimens did not allow us to evaluate their specific effects, however, the similar average dose of Valporate, an ASM commonly used in JME and known to negatively affect gait towards an extrapyramidal pattern (Jamora et al., 2007; Onofrj et al., 1998), argues against the contribution of specific ASM compositions to the observed differences. Future studies with larger cohorts are needed to better understand the effects of polytherapy in JME patients. In addition, prospective study of drug‐naïve patients is needed in order to finally rule out a general drug effect that degrades gait performance.

## 5 CONCLUSIONS

Altogether, this is the first study to report on gait alterations in both simple and challenging walking in DR‐JME patients. Abnormal electrical activity in the postcentral, precentral and superior frontal gyri provide evidence to large neural networks involvement in JME. These clinical and electrophysiological findings are of importance as they offer potential biomarkers for disease severity and drug responsiveness in JME. Such biomarkers are essential as they could help in counseling patients and families regarding the expected trajectory of the disease, aiming for implementing precision medicine approaches. Our findings suggest that the assessment of gait, a complex motor function, yet a very readily available neurological testing, can add valuable information to the clinical management of JME patients.

## AUTHOR CONTRIBUTIONS

IM, LG, NG, JH, AM, and FF contributed to the conception and design of the study; MY, SG, SN, and IM contributed to the acquisition and analysis of data; IM and FF contributed to drafting the text or preparing the figure.

## CONFLICT OF INTEREST

None of the authors has any conflict of interest to disclose.

### PEER REVIEW

The peer review history for this article is available at https://publons.com/publon/10.1002/brb3.2872.

## Supporting information

Supplementary Table S1

## Data Availability

Data will be made available by request from any qualified investigator.
