# Supplementary material for: Abnormal gait and motor cortical processing in drug‐resistant juvenile myoclonic epilepsy
Source: Brain Behav. 2023 Jan 5;13(2):e2872. doi: 10.1002/brb3.2872 (PMC9927833; doi:10.1002/brb3.2872)
Supplement: Supplementary file 1 — Supplementary Table S1 [file BRB3-13-e2872-s001.docx]

**SUPPLEMENTARY**

| Sub | group | Current anti-epileptic medication |
| --- | --- | --- |
| 001 | respondent | depalept chrono, valproic acid |
| 002 | respondent | Clonex, Topamax, lamictal |
| 007 | respondent | lamictal |
| 008 | respondent | Topiramate (Topamax), vimpat, Clonex |
| 009 | respondent | lamictal |
| 011 | respondent | briviact |
| 012 | respondent | depalept chrono, keppra |
| 013 | respondent | lamictal, DEPALEPT Chrono |
| 014 | respondent | Depalept, keppra, Clonex |
| 016 | respondent | Depalept |
| 017 | respondent | Keppra |
| 004 | resistance | Tegretol, keppra, vimpat, frisium |
| 005 | resistance | vimpat, frisium, Epanutin, uramox, cannabis |
| 006 | resistance | depalept chrono, cannabis |
| 010 | resistance | lamictal, keppra |
| 015 | resistance | lamictal, Clonex |
| 018 | resistance | Keppra, Depalept, Clonex |
| 019 | resistance | Missing |
| 003 | resistance | lamictal (Lamogine), keppra, frisium, depalept chrono, cannabis |

**Supplementary Table 1**
